# Mesoporous Silica with Site-Isolated Amine and Phosphotungstic Acid Groups: A Solid Catalyst with Tunable Antagonistic Functions for One-Pot Tandem Reactions**

**DOI:** 10.1002/anie.201101449

**Published:** 2011-09-15

**Authors:** N Raveendran Shiju, Albert H Alberts, Syed Khalid, David R Brown, Gadi Rothenberg

**Affiliations:** Van't Hoff Institute for Molecular Sciences, University of AmsterdamScience Park 904, 1098 XH Amsterdam (The Netherlands); Materials and Catalysis Research Centre, Department of Chemical and Biological Sciences, University of HuddersfieldQueensgate HD1 3DH (UK); National Synchrotron Light Source, Brookhaven National LaboratoryUpton, NY 11973 (USA)

**Keywords:** heterogeneous catalysis, mesoporous materials, NMR spectroscopy

Scientists engaged in heterogeneous catalysis often cite enzymes as their model catalysts. Enzymes can efficiently catalyze multistep processes that give various types of biomolecules.^1^ Remarkably, many enzymes combine two antagonistic catalytic functions, such as acid and base functions.^2^ Attracted by this challenge, several groups synthesized homogeneous catalysts that can successfully combine chemically hostile functions.^3^ However, the difficulty lies in controlling the separation between these groups and simultaneously working at practical catalyst concentrations. In principle, both of these problems can be solved using heterogeneous catalysis. But synthesizing solid catalysts that combine hostile functions is no easy task. Not surprisingly, examples of solids with both acidic and basic functions are limited. The reported examples combine acids such as sulfonic acids, silanols, ureas, and thiols with amines.^4^ However, problems such as low catalytic activity because of weak acidity, complicated preparation, and lack of a continuous range of acidic and basic catalytic sites often significantly limit their application in organic reactions.

Here, we present a simple and straightforward route to such bifunctional solids. We combine amine base functions and heteropolyacid functions on periodic mesoporous silica, obtaining an efficient and robust bifunctional acid–base solid catalyst. This new material enables one-pot cooperative catalysis. Moreover, the synthesis permits easy tailoring of the acid/base properties by controlling the number and surface concentration of the acid and base sites, respectively. We demonstrate the catalytic efficiency and robustness of this new system in two cascade reactions: a tandem deprotection–Henry reaction, and a tandem deprotection–aldol reaction. Excellent yields and selectivities are obtained in both cases.

Figure 1 shows the synthesis of the catalyst. We started by making a mesoporous silica support **S** (Figure 1, top; pore diameter of 4.9 nm and surface area of 870 m^2^ g^−1^, see the Experimental Section). The base functions were then added by grafting 3-aminopropyl groups, giving the base catalyst **SB** (Figure 1, middle). This material was then immersed in a methanolic solution of phosphotungstic acid (H_3_PW_12_O_40_). The 3-ammoniumpropyl groups immobilized the acid polyanions, creating the acid/base catalyst **SAB** (Figure 1, bottom). By controlling the ratio of immobilized heteropolyacids and aminopropyl tethers, we succeeded in reacting only part of the amino groups, creating bifunctional catalytic sites inside the silica mesopores.

**Figure 1:**
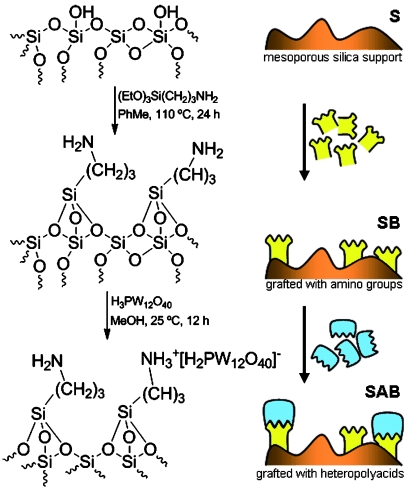
Catalyst synthesis.

The catalysts were characterized before and after each functionalization step. Figure 2 shows the N_2_ adsorption isotherms of **S** and **SAB**. Both materials are mesoporous, with average pore diameters of 4.9 and 3.4 nm, respectively. The isotherms show that grafting the base and immobilizing the acid do not change the structure. **SAB** adsorbs less nitrogen than **S**, because a fraction of the pores is already occupied by the aminopropyl and phosphotungstic groups. However, the pores are still wide enough to allow easy diffusion of reactant and product molecules. The structure retention after immobilization was also confirmed by X-ray diffraction (see the Supporting Information).

**Figure 2:**
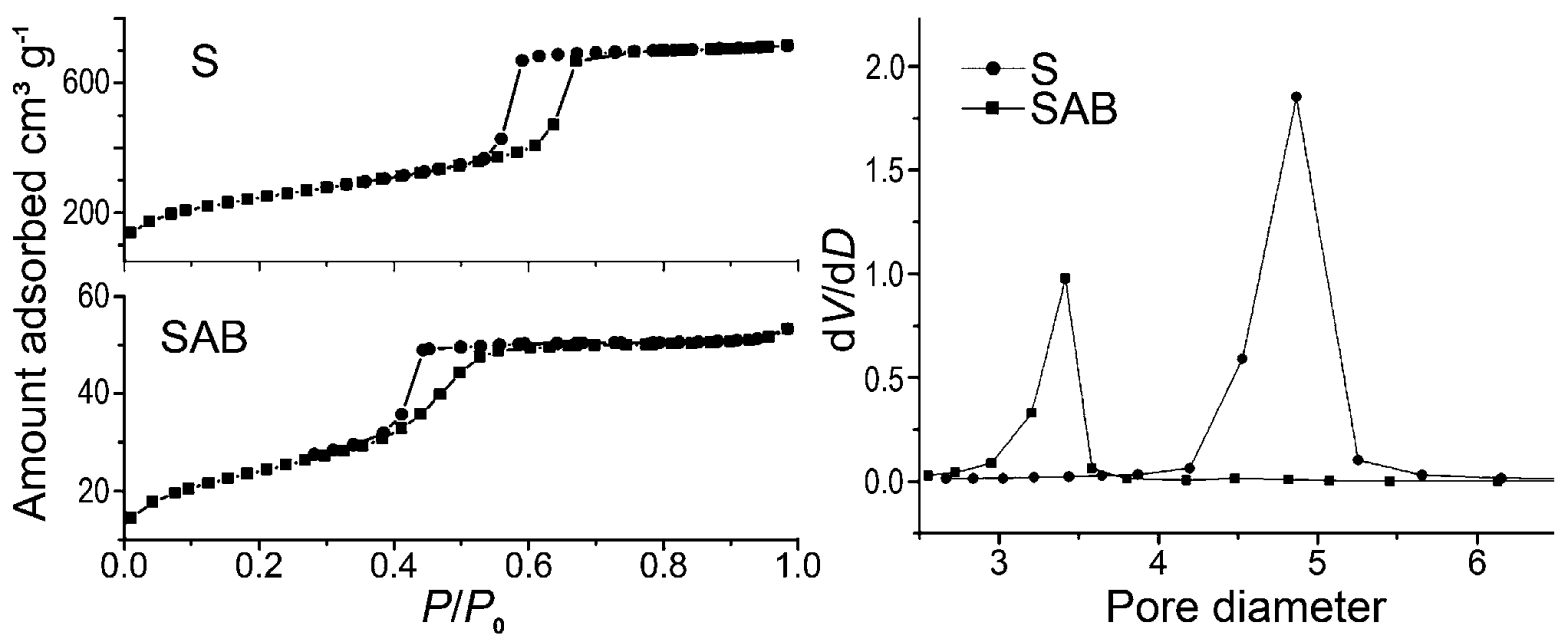
N_2_ adsorption isotherms at standard temperature and pressure (left) and pore-size distribution (right) of **S** and **SAB**.

^13^C cross-polarization (CP) magic angle spinning (MAS) NMR spectra showed peaks at 11, 22, and 43 ppm (Figure 3). These peaks are typical for the SiCH_2_CH_2_CH_2_NH_2_ chain, indicating the successful grafting of the aminopropyl groups on silica support **S**. The presence of T^3^ and T^2^ functionalities in the ^29^Si CP-MAS NMR spectra (Figure 4) confirmed a strong covalent linkage between the organic groups and the silica surface (see also Figure S3 in the Supporting Information). A comparison of the W-L_3_ edge region in the X-ray absorption near-edge structure (XANES) spectrum of **SAB** with that of bulk phosphotungstic acid showed no major shifts in position and amplitude. This confirms the absence of geometrical or electronic changes in the immobilized groups (Figure 5). Moreover, ^31^P NMR spectroscopy also showed that the Keggin structure of the phosphotungstic acid was maintained after immobilization (singlet at −15.5 ppm, see the Supporting Information).

**Figure 3:**
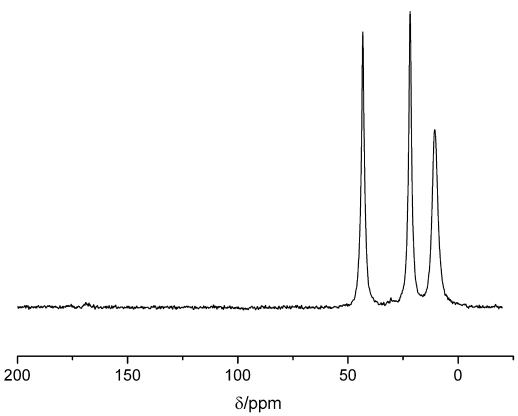
^13^C CP-MAS NMR spectrum of **SAB**, indicating successful grafting of the aminopropyl groups on **S.**

**Figure 4:**
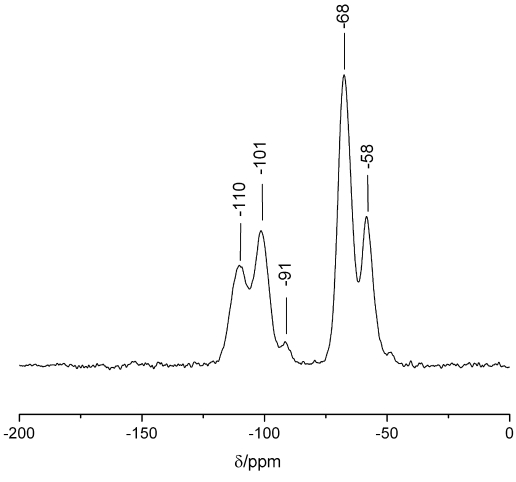
^29^Si CP-MAS NMR spectrum of **SAB.** Peaks at −110, −101, −91, −68, and −58 ppm were assigned to the Q^4^ (Si(OSi)_4_), Q^3^ (Si(OH)(OSi)_3_), Q^2^ (Si(OH)_2_(OSi)_2_), T^3^ (SiR(OSi)_3_), and T^2^ (Si(OH)R(OSi)_2_) sites, respectively.

**Figure 5:**
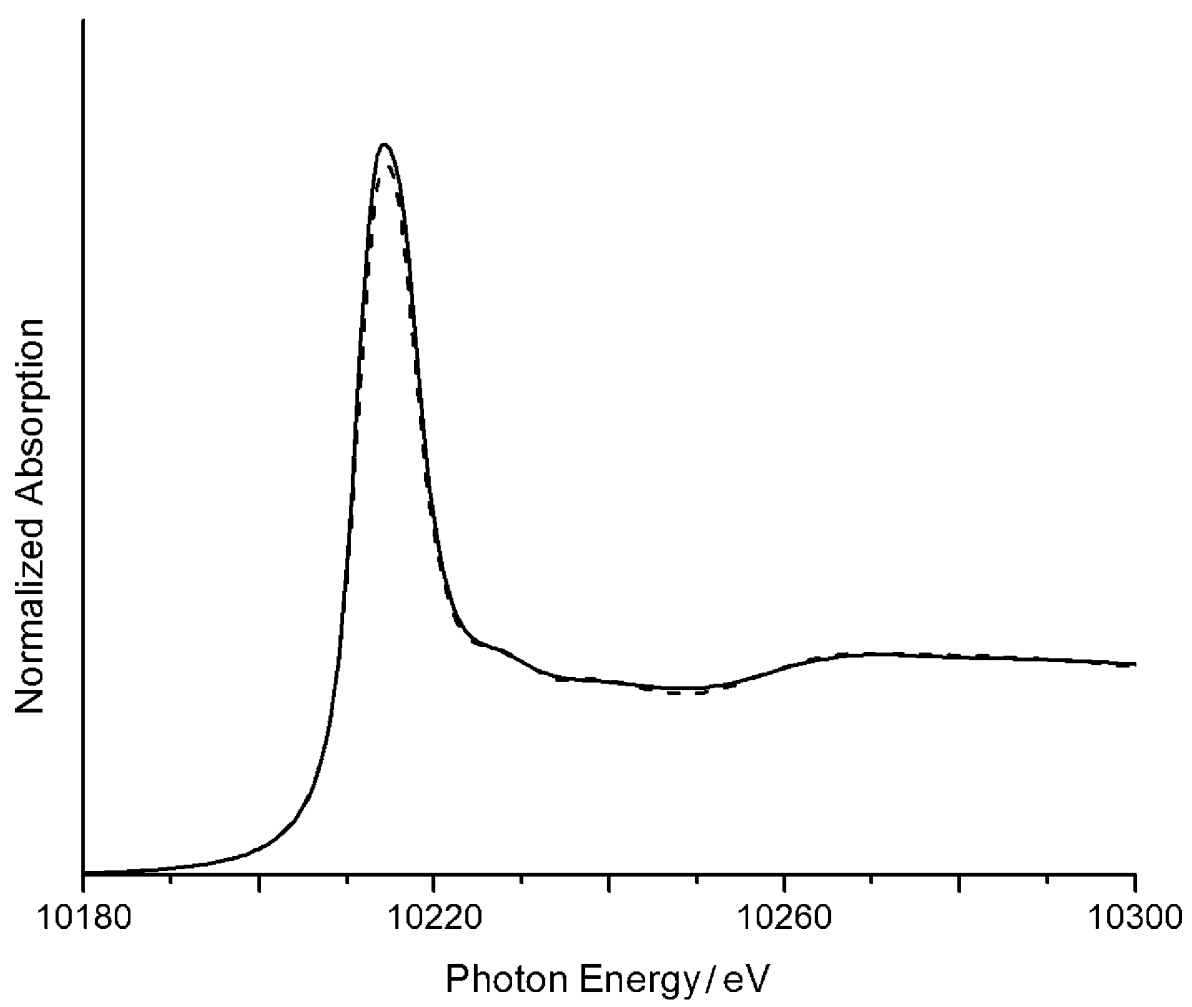
The tungsten L_3_ edge XANES spectra of **SAB** (straight line) and bulk phosphotungstic acid (dotted line), indicating retention of the Keggin structure after immobilization.

We then investigated the bifunctionality of catalyst **SAB** in the one-pot tandem conversion of dimethoxymethylbenzene (benzaldehydedimethylacetal) **1** to *trans*-1-nitro-2-phenylethylene **3** (Scheme 1; the functionality that catalyzes each step is shown in blue). The first step of this reaction is the acid-catalyzed deacetalization of **1** to benzaldehyde **2**. In the second step, which is base-catalyzed, the benzaldehyde reacts with nitromethane, giving the nitro product **3**. Table 1 shows the results. High conversions and yields were observed in the presence of the **SAB** catalyst (entry 1 in Table 1). When **SB** was used instead of **SAB**, no reaction occurred with **1** (entry 2 in Table 1). However, control reactions starting from **2** confirmed that **SB** is an excellent base catalyst for the second step, converting benzaldehyde with nitromethane to **3** in near-quantitative yield (entry 4 in Table 1). These results show that both the amino groups and the acid polyanions in **SAB** retain their reactivity. The immobilized polyanions, which are still acidic, catalyze the deacetalization of **1** to **2** (Figure 6). This is followed by the Henry reaction of **2** with nitromethane at the free amino groups. The key requirement for the tandem deacetalization–nitroaldol reaction is the ratio between amino groups and polyanions. If all the amino groups are used for polyanion immobilization, the reaction stops at the first step and benzaldehyde is the sole product (entry 3, Table 1). In our bifunctional catalyst, only part of the amine groups are used for immobilizing polyacids; the remaining half are free. We also confirmed that immobilization does not affect the intrinsic activity of polyacid groups by comparing the catalytic activities of **SAB** and bulk phosphotungstic acid in ethyl acetate hydrolysis reaction. The catalytic activities, calculated based on the amount of acidic protons (measured by pyridine adsorption), were similar (102 and 95 mmol mol^−1^ min^−1^).

**Figure 6:**
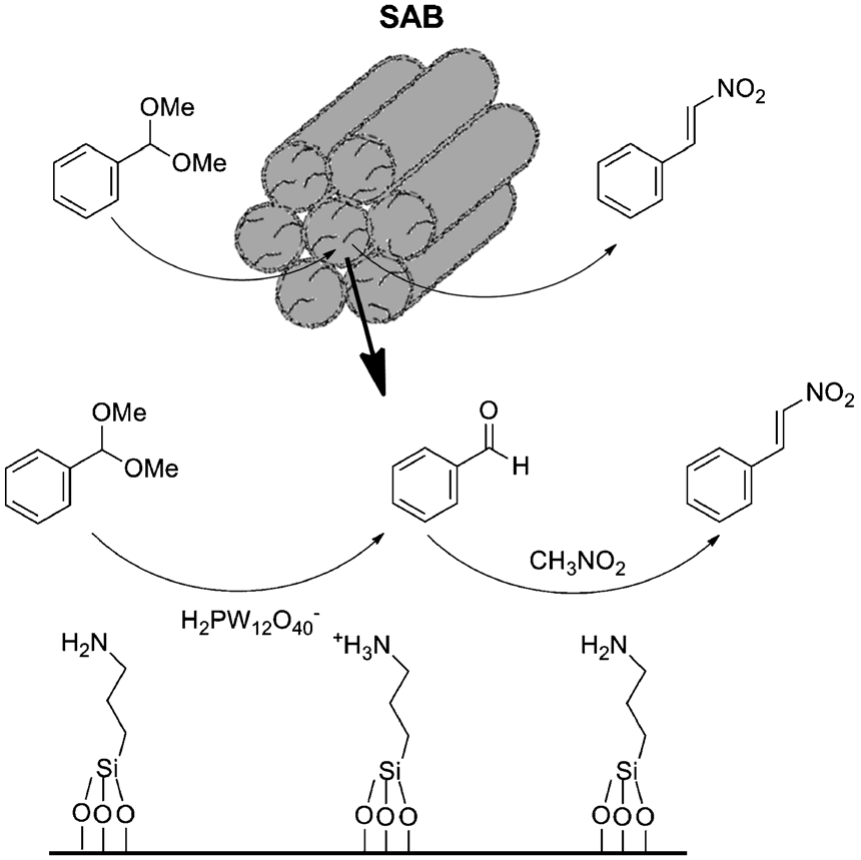
The conceptual diagram showing the bifunctional catalysis by free amino groups and phosphotungstic acid immobilized inside the SBA-15 nanoreactors.

**Scheme 1:**
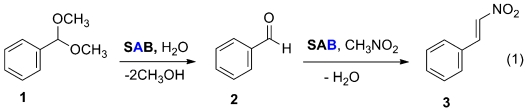
One-pot tandem conversion of benzaldehydedimethylacetal **1** to *trans*-1-nitro-2-phenylethylene **3.** The acid sites of **SAB** catalyze the deacetalization first and the base sites then catalyze the Henry condensation.

**Table 1:** Tandem deprotection–Henry reaction.[a]

Entry	Catalyst	Substrate	Conversion [%]	Yield GC/isolated [%]	
				**2**	**3**
1	**SAB**[b]	**1**	≈98	tr	97/92
2	**SB**	**1**	–	–	–/–
3	**SAB**Table [c]	**1**	≈99	95/–	5/–
4	**SB**[e]	**2**	>99	–	>99/91
5	**S**	**1**	tr	tr	tr
6	**SAB**[d]	**1**	95	tr	94/90

[a] Reaction conditions: benzaldehyde dimethyl acetal (1 mmol), CH_3_NO_2_ (10 mL), 50 °C, 12 h.

[b] Phosphotungstic acid (HPW)/aminopropyl (AP) ratio around 0.5:1 (molar).

[c] HPW/AP ratio around 1:1 (molar).

[d] Fourth recycle of the catalyst of entry 1.

[e] For Henry reaction alone starting from entry 2. tr: trace.

Control reactions confirmed that **S** alone did not catalyze the reaction of **1** under the same conditions (entry 5, Table 1). Moreover, the **SAB** catalyst was easily recovered by filtration and reused several times without the loss of the catalytic activity and selectivity (entry 6, Table 1; the reactions stopped as soon as the catalyst was filtered off the hot reacting mixture). This rules out the possibility of leaching of active species into the reaction solution.

Similarly, we evaluated the **SAB** catalyst in the tandem reaction of **1** through **2** to benzylidene malononitrile **4** (Scheme 2). Following the first deacetalization step, **2** undergoes a base-catalyzed aldol condensation with malononitrile. **SAB** efficiently catalyzed this tandem deacetalization–aldol reaction as well, which yielded 91 % of **4** and shows the versatility of our catalyst (Table 2). Control reactions in the presence of homogeneous *p*-toluenesulfonic acid gave no product because of the neutralization of the amino groups of the catalyst.

**Scheme 2:**
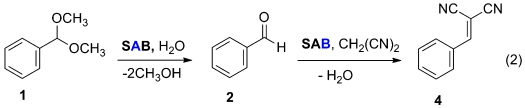
Tandem deacetalization–aldol reaction of dimethoxymethylbenzene **1** through **2** to benzylidene malononitrile **4.**

**Table 2:** Tandem deprotection–aldol reaction[a]

Entry	Catalyst	Substrate	Conversion [%]	Yield GC/isolated [%]	
				**2**	**4**
1	**SAB**[b]	**1**	>98	tr	99/91
2	**SB**	**1**	–	–	–/–
3	**SAB**[c]	**1**	≈99	≈95/–	5/–
4	**SB**[e]	**2**	>99	–	>99/92
5	**S**	**1**	tr	tr	tr
6	**SAB**[d]	**1**	95	tr	≈95/91

[a] Reaction conditions: benzaldehyde dimethyl acetal (1 mmol), CH_2_(CN)_2_ (10 mL), 50 °C, 12 h.

[b] HPW/AP ratio around 0.5:1 (molar).

[c] HPW/AP ratio around 1:1 (molar).

[d] Fourth recycle of the catalyst in entry 1.

[e] For the aldol reaction alone starting from 2. tr: trace.

In conclusion, we showed that phosphotungstic acid immobilized on amine-grafted mesoporous silica is an efficient bifunctional catalyst. The catalyst combines two antagonistic active functions on one solid catalyst, allowing acid–base tandem conversions in a single pot. We can prepare this catalyst as well as tune it easily. The catalyst can be made predominantly basic, or predominantly acidic, or equally acidic and basic by changing the ratio of polyacid and amine groups. Similar types of catalysts can be synthesized using other heteropolyacids, which further widens the scope of these materials.

## Experimental Section

Catalyst synthesis: Mesoporous silica SBA-15 (**S**) was synthesized by route using block copolymer templates following a literature report.^5^ Pluronic P123 (average molecular weight of 5800, EO20PO70EO20, BASF; 4.0 g) was dissolved in a solution of distilled water and 2 m HCl (125 and 25 g, respectively) and stirred at 25 °C. After stirring for 3 h, tetraethyl orthosilicate (TEOS, Aldrich; 8.6 g) was added slowly under stirring. The solution was vigorously stirred at 40 °C for 24 h. The mixture was aged at 80 °C for 48 h. The resulting white solid was filtered off, washed, and air-dried at room temperature for 24 h. The sample was calcined in air at 550 °C for 5 h.

SBA-15 was heated at reflux with 3-aminopropyltriethoxysilane in dry toluene for 24 h to obtain aminopropyl-grafted mesoporous silica (**SB**). The amount of grafted amino groups were determined by elemental analysis.

Immobilization of heteropolyacids: **SB** was stirred with a methanolic solution of required amounts of phosphotungstic acid (Aldrich) based on grafted amino groups for 12 h. The solid was then washed in warm water (333 K), followed by drying to obtain the bifunctional catalyst **SAB**.

Characterization of the catalyst: Powder X-ray diffraction patterns were collected on a Bruker powder X-ray diffractometer (D5000) operated at 40 kV and 20 mA using nickel-filtered Cu K_α_ radiation (1.5406 Å). N_2_ adsorption–desorption isotherms were measured at 77 K on a micromeritics ASAP-2000 after evacuation at 473 K for 5 h. The surface areas and the average pore sizes were calculated by the Brunauer–Emmett–Teller (BET) and Barrett–Joyner–Halender (BJH) methods, respectively. The solid-state ^13^C and ^29^Si NMR spectra were obtained using a Varian VNMRS spectrometer operating at 100.56 mhz for ^13^C and 79.44 mhz for ^29^Si equipped with a 6 mm (outside diameter of the rotor) MAS probe. The spectral referencing is with respect to neat, external tetramethylsilane.^31^P NMR spectra were obtained with a Varian VNMRS instrument operating at 161.88 mhz. A direct polarization pulse sequence was used to record the spectra, with a long recycle (300 s). The spectra were referenced with respect to 85 % H_3_PO_4_.

The powder X-ray diffraction pattern of the calcined material (**S**) shows the typical pattern of SBA-15 (Figure S1 in the Supporting Information). The retention of the SBA-15 structure after immobilization was also confirmed by X-ray diffraction (see Figure S2a in the Supporting Information). A major signal at −15.5 ppm was observed in the ^31^P NMR spectrum of **SAB** (see Figure S2b in the Supporting Information). Phosphotungstic acid (HPW) has a symmetrical cubic structure with a central P atom.^6^ The signal at −15.5 ppm confirms that the Keggin structure of the HPW was maintained after immobilization.^6^ There is a minor peak at −13.7 ppm, which may be due to distortion of the HPW cubic structure because of the functionalization. However, this is only a negligible fraction of the immobilized HPW.

X-ray absorption spectra around the W-L_3_ edge were recorded at the X18 A beam line at the National Synchrotron Light Source, Brookhaven National Laboratory. We used a Si(111) double-crystal monochromator. The energy was calibrated using a W foil.

Catalytic testing: The reactions were carried out in liquid phase in a 50 mL glass reactor equipped with a condenser and a magnetic stirrer. Benzaldehyde dimethylacetal (1 mmol) and nitromethane (10 mL) were stirred at 50 °C under N_2_ with 30 mg of the catalyst in powder form. To monitor the reaction, samples of the reaction mixture were taken periodically and analyzed by gas chromatography (GC, Perkin–Elmer Clarus 500) using a 50 m BP5 capillary column and an free induction decay (FID) detector. After the completion of the reaction, the catalyst was separated by filtration. The filtrate was analyzed by GC and the products were confirmed by GC-MS and ^1^H NMR spectroscopy. The recovered catalyst was washed with nitromethane and acetone, and was then reused for the above one-pot reaction. Deacetalization–aldol reaction of benzaldehyde dimethylacetal and malononitrile was also conducted in a similar way. Ethyl acetate hydrolysis was carried out in a round bottom flask, fitted with a condenser and a magnetic stirrer. A dilute aqueous solution of ethyl acetate (10 wt %) was stirred with catalyst powder (2 wt %) at 343 K and the catalytic activity was measured by GC.

## Supporting information

Detailed facts of importance to specialist readers are published as ”Supporting Information”. Such documents are peer-reviewed, but not copy-edited or typeset. They are made available as submitted by the authors.
