# Supplementary material for: Mesoporous Silica with Site-Isolated Amine and Phosphotungstic Acid Groups: A Solid Catalyst with Tunable Antagonistic Functions for One-Pot Tandem Reactions
Source: Angew Chem Int Ed Engl. 2011 Sep 15;50(41):9615–9. doi: 10.1002/anie.201101449 (PMC3303888; doi:10.1002/anie.201101449)
Supplement: Supplementary file 1 [file anie0050-9615-SD1.pdf]

Angewandte Chemie
Eine Zeitschrift der Gesellschaft Deutscher Chemiker

Supporting Information
© Wiley-VCH 2011
69451 Weinheim, Germany

# Mesoporous Silica with Site-Isolated Amine and Phosphotungstic Acid Groups: A Solid Catalyst with Tunable Antagonistic Functions for One-Pot Tandem Reactions**

*N. Raveendran Shiju,* Albert H. Alberts, Syed Khalid, David R. Brown, and Gadi Rothenberg**

anie_201101449_sm_miscellaneous_information.pdf

**Supporting Information**

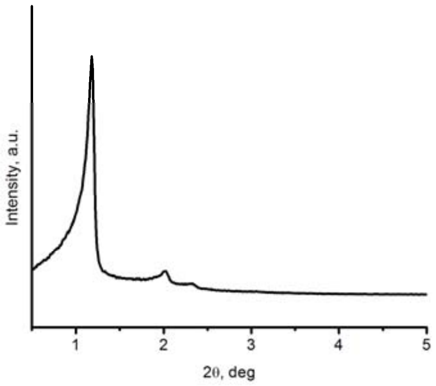


***Figure S1.*** Powder X-ray diffraction pattern of calcined SBA-15.

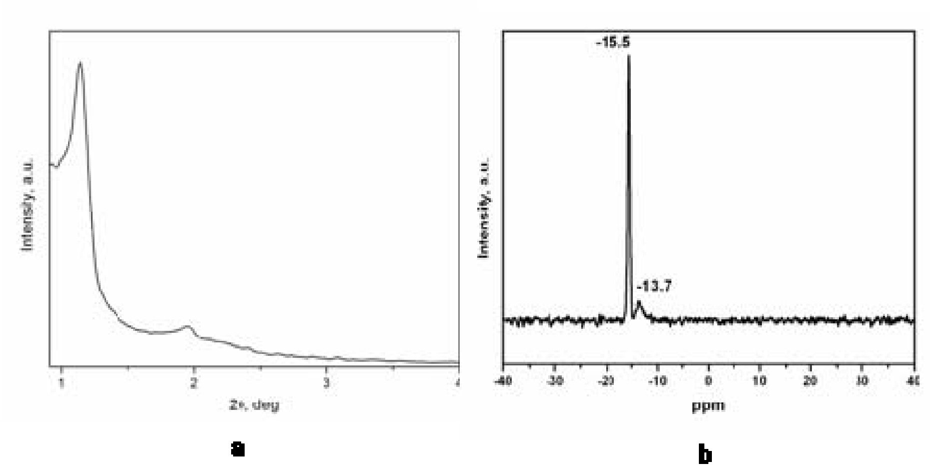


***Figure S2***. Powder X-ray diffraction pattern (a) and $^{31}$P NMR spectrum (b) of **SAB**.

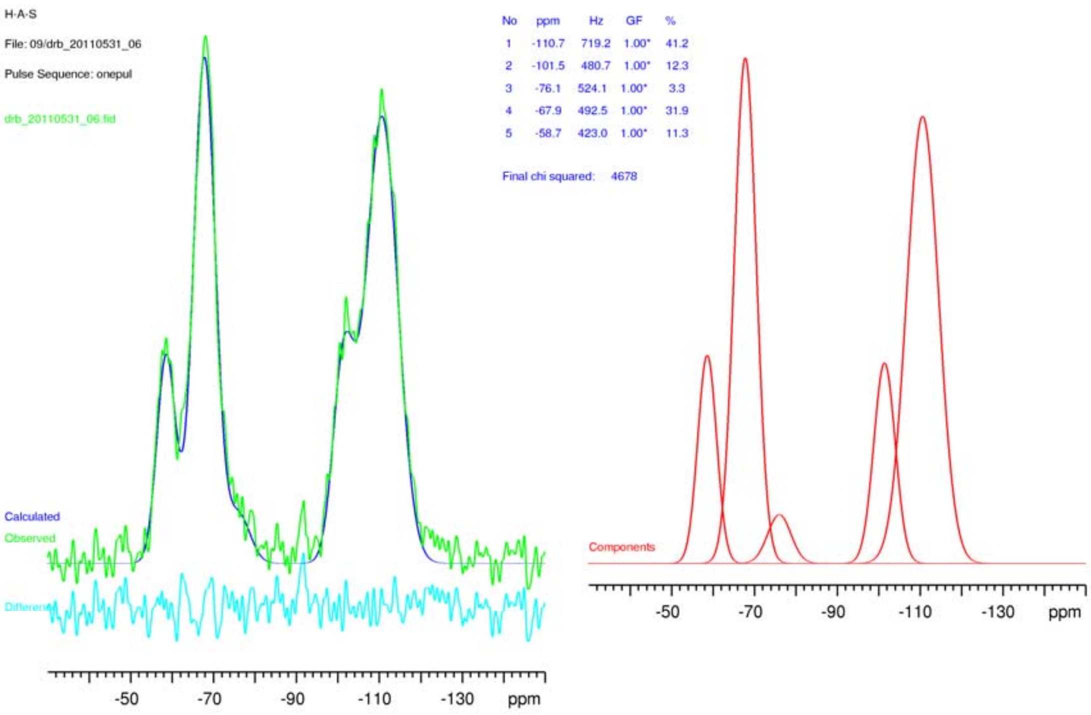


***Figure S3***. Direct excitation silicon MAS NMR spectrum of **SAB**. The spectrum was recorded with a 60 s recycle and the deconvolution shows relative intensities of different peaks.
